# Correlates of psychological intimate partner violence with HIV care outcomes on patients in HIV care

**DOI:** 10.1186/s12889-021-11854-x

**Published:** 2021-10-09

**Authors:** R. J. Fredericksen, R. M. Nance, B. M. Whitney, B. N. Harding, E. Fitzsimmons, C. Del Rio, J. Eron, D. J. Feaster, A. S. Kalokhe, W. C. Mathews, K. H. Mayer, L. R. Metsch, M. J. Mugavero, J. Potter, C. O’Cleirigh, S. Napravnik, B. Rodriguez, S. Ruderman, Delaney JAC, H. M. Crane

**Affiliations:** 1 Department of Medicine, University of Washington, Seattle, Washington USA; 2 Department of Global Health, Emory University, Atlanta, Georgia; 3 School of Global Public Health, University of North Carolina, Chapel Hill, North Carolina USA; 4 Department of Public Health Sciences, University of Miami, Miami, Florida USA; 5 Department of Medicine, Emory University, Atlanta, Georgia; 6 Department of Medicine, University of California – San Diego, San Diego, California USA; 7 The Fenway Institute, Boston, MA USA; 8 Department of Sociomedical Sciences, Columbia University, New York, NY USA; 9 Department of Medicine, University of Alabama – Birmingham, Birmingham, AL USA; 10 Department of Medicine, Harvard University, Cambridge, MA USA; 11 Department of Psychiatry, Massachusetts General Hospital, Boston, MA USA; 12 Department of Epidemiology, University of North Carolina, Chapel Hill, North Carolina USA; 13 Department of Medicine, Case Western Reserve University, Cleveland, OH USA; 14 College of Pharmacy, University of Manitoba, Winnipeg, Manitoba USA

**Keywords:** Psychological violence, HIV care, Patient reported outcomes

## Abstract

**Background:**

Among people living with HIV (PLWH), physical intimate partner violence (IPV) is associated with poor virologic, psychiatric, and behavioral outcomes. We examined non-physical, psychological intimate partner violence (psy-IPV) and HIV care outcomes using data from two U.S. consortia.

**Methods:**

We conducted multivariable analyses with robust standard errors to compare patients indicating/not indicating psy-IPV.

**Results:**

Among PLWH (*n* = 5950), 9.5% indicated psy-IPV; these individuals were younger (− 3; 95% CI [− 2,-4], *p*-value < 0.001), less likely to be on antiretroviral treatment (ART) (0.73 [0.55,0.97], *p* = 0.03), less adherent to ART (− 4.2 [− 5.9,-2.4], *p* < 0.001), had higher odds of detectable viral load (1.43 [1.15,1.78], *p* = 0.001) and depression (2.63 [2.18,3.18], *p* < 0.001), and greater use of methamphetamines/crystal [2.98 (2.30,3.87),*p* < 0.001], cocaine/crack [1.57 (1.24,1.99),*p* < 0.001], illicit opioids [1.56 (1.13,2.16),*p* = 0.007], and marijuana [1.40 (1.15,1.70), *p* < 0.001].

**Conclusion:**

Psychological IPV, even in the absence of physical or sexual IPV, appears to be associated with HIV care outcomes and should be included in IPV measures integrated into routine HIV care.

**Supplementary Information:**

The online version contains supplementary material available at 10.1186/s12889-021-11854-x.

## Background

Intimate partner violence (IPV) is defined as physical, sexual, and/or psychological violence by a current or former intimate partner [1]. Psychological IPV (psy-IPV) is defined as the use of verbal and non-verbal communication with the intent to harm another person mentally or emotionally, and/or to exert control over another person [1]. IPV is known to disproportionately affect women, and men who have sex with men (MSM); lifetime global prevalence of physical or sexual IPV against ever-partnered women is estimated at 27% with a past 12-month prevalence of 13% (25 and 6% respectively for North America) [2]; in the U.S., men who have sex with men (MSM) are estimated to have physical and sexual IPV rates similar to heterosexual women [3]. Psy-IPV estimates suggest a rate of ~ 47% [4, 5]. Studies among people living with HIV (PLWH), among women and MSM, show high and wide ranging lifetime physical IPV (26–62%/15–39%), sexual IPV, (22–44%/8–33%), and psy-IPV (55%/22–73%) [6]. Among substance-using PLWH, lifetime rates of all types of IPV have been found to be very high: 56% among cocaine/crack-using PLWH in the southern U.S., with the highest rates among women and gay men (68 and 71%) [7].

To date, there has been a lack of robust systematically-collected data on IPV rates across large populations of PLWH. Living with HIV has been associated with both psychological and physical IPV [8], and any IPV among PLWH has been associated with poorer antiretroviral treatment (ART) adherence, more clinically relevant interruptions in care [7, 9–11], and increased HIV-related hospitalizations [11]. In addition, in both PLWH and those without HIV, any IPV has been associated with psychiatric conditions, such as depression [6, 12–15] and post-traumatic stress disorder (PTSD) [6, 16] and adverse health behaviors, including substance abuse [6, 12, 17–20] and high-risk sexual behavior [4, 6, 21, 22]. There appears to be a link between IPV and immune defenses. Among HIV-negative women at high risk for contracting HIV, both lifetime and past-year violence of any type was associated with increased CD4+ activation [23]; among PLWH, physical IPV has been shown to have a negative impact on virologic outcomes [24] and CD4+/CD8+ T-cell decay [25].

Psychological IPV is less well-studied. However, it appears to be more common than physical or sexual IPV [26–28], and, in a longitudinal study of women, more likely to continue in the absence of physical or sexual IPV [29]. Stress induced by psy-IPV has an impact similar to physical IPV on immune functioning [23, 24] and a negative impact on an array of health behaviors and outcomes [16, 30]. One study suggested that psy-IPV has been associated with a detectable viral load, CD4 < 200, and high no-show rate for HIV care visits [24]. However, this study was limited by a small sample size at a single site [24]. A longitudinal study of young ART-naïve women with HIV in South Africa found psy-IPV in the form of emotional abuse was associated with a faster decline in markers of cellular immunity even when controlling for exposure to physical or sexual IPV [25]. In HIV-uninfected populations, psy-IPV has been associated with adverse mental health and behavioral outcomes including depression severity [31], post-traumatic stress symptom severity [31], substance use [32], and sexual risk behavior [4], the latter even when controlling for physical and sexual violence [16]. Outcomes and factors such as substance use, HIV, and violence, have been described as a syndemic [33]; these, along with mental health impacts, appear inter-related and synergistic [33]. For example, avoidance behavior associated with PTSD has been found to mediate the relationship between psy-IPV and substance use, depression severity, and sexual risk behavior [16, 31].

Most studies of psy-IPV have been limited in scope in terms of both population and outcomes, with most focusing exclusively on women [16, 23, 25, 30]. We sought to better understand the correlates and impact of psy-IPV in a large geographic, clinical, and gender-diverse sample of PLWH on HIV care continuum outcomes and health behaviors. Building on prior work among women in the general population or small studies of PLWH [23, 25], we hypothesized that psy-IPV will inhibit viral suppression/CD4. In addition, as noted in populations having experienced physical IPV, we hypothesized psy-IPV will worsen ART adherence among PLWH, [7, 9, 10] increase depressive symptoms [12–14], and increase substance use [12, 17–20].

## Methods

### Study population

This cross-sectional study included PLWH from two large collaborations in the U.S. including Puerto Rico to ensure demographic, geographic, and clinical diversity, with PLWH from geographically dispersed clinical care settings to enhance generalizability and study settings focused on substance using PLWH to ensure inclusion of vulnerable populations. Centers for AIDS Research Network of Integrated Clinical Sites (CNICS) (https://www.uab.edu/cnics/) is a cohort study of PLWH in clinical care at eight sites across the US from 1995 to present [34]. Seek, Test, Treat, Retain (STTR) is a consortium initiated by the National Institute on Drug Abuse [35, 36] to address research questions related to HIV care continuum outcomes among vulnerable particularly substance using populations.

### Study subjects

From CNICS, we included PLWH representing six CNICS sites: the 1917 Clinic at University of Alabama-Birmingham; Case Western Reserve University in Cleveland, OH; Fenway Community Health-Boston, MA; Owen Clinic at University of California at San Diego; University of North Carolina; and Madison Clinic at Harborview Medical Center/University of Washington-Seattle. IPV data collection from these patients began in 2016. From the STTR study consortium, we included PLWH from two studies: Proyecto PACTo (Proveyendo Acceso a Cuidado y Tratamiento) [37], and Project RETAIN [38].

Proyecto PACTo is a clinical trial which evaluated effectiveness of the “Enhanced HIV Care Access and Retention Intervention” in achieving HIV virologic suppression among substance using PLWH in Puerto Rico that ran from 2013 to 2014. Project RETAIN is a clinical trial done to evaluate the efficacy of an integrated “Retention Clinic” in achieving virologic suppression among cocaine/crack-using PLWH in Florida and Georgia that ran from 2013 to 2015.

For both the STTR (PACTO/RETAIN) and CNICS groups, our analyses included PLWH who were 18 years of age or older and completed a self-administered patient-reported outcome (PRO) measure querying IPV. For PACTO/RETAIN, we included PLWH who answered IPV questions at their baseline visit. For CNICS, we included PLWH that had been administered the IPV measure in their most recent PRO assessment at the beginning of their routine care visit. Table 1 shows full inclusion and exclusion criteria across consortia and networks.

**Table 1:** Psy-IPV study population: inclusion/exclusion criteria, data collection dates, and regions

Consortium	Inclusion criteria	Exclusion criteria	Data collection dates	Regions
CNICS	≥18	Unable or unwilling to complete questionnaire	2016-2018	AL, CA, MA, NC, OH, WA
	PLWH			
	In routine HIV care			
	English or Spanish-speaking			
PACTO (STTR)	≥18	Unable to consent due to cognitive or developmental impairment	2013-2014	Puerto Rico
	PLWH			
	Able to communicate in English			
	Report any drug (excluding nicotine) and/or heavy alcohol use within the past 12 months			
RETAIN (STTR)	≥18	Unable to consent due to cognitive or developmental impairment	2013-2015	FL, GA
	PLWH			
	Cocaine/crack use in past 3 months			
	AIDS-defining illness OR CD4 <350 AND a viral load >1000 copies/mL in the medical record in the past 3 months, OR Have a CD4 count <350 cells/uL AND a viral load >200 copies/mL as obtained via baseline blood draw, OR Clinical profile indicative of a persistently detectable HIV viral load (>200 copies/mL)	Currently receiving patient navigator services for HIV care or substance use treatment		

### Data sources

Both CNICS and STTR have data repositories that harmonize and integrate demographic, clinical, laboratory, and other data such as patient-reported measures including IPV [36, 39].

### Psychological intimate partner violence measures

CNICS utilizes a brief validated measure, the IPV-4 [40], that is inclusive of physical IPV, sexual IPV, and two forms of psy-IPV (see Table 2): controlling behavior by an intimate partner and fearfulness of a partner in the past year. The IPV-4, initiated in 2016, is administered to PLWH within CNICS clinics on-site prior to their clinic visit as part of routine care. The PACTO and RETAIN studies used the second two items of a lifetime IPV measure known as STaT [an acronym for ‘slapped, threatened, and throw (things)’], as well as an item querying controlling behavior that was used in development of the STaT measure [41]; these items were adapted to reflect a 6 month recall period (Table 2). Data from items regarding controlling behavior were harmonizable across CNICS and PACTO/RETAIN (in Table 2, CNICS item 1 harmonizes with PACTO/RETAIN item 3), as were data regarding threatening behavior (in Table 2, CNICS item 2 harmonizes with PACTO/RETAIN items 1 and 2); these two areas comprise our dimensions of psy-IPV. Psy-IPV was considered reported if a person answered “yes” to any of these items.

**Table 2:** Psychological intimate partner violence measures

CNICS:	
1. In the PAST YEAR, did a current or former partner… Make you feel cut off from others, trapped, or controlled in a way you did not like? 2. In the PAST YEAR, did a current or former partner… Make you feel afraid that they might try to hurt you in some way?	
PACTO and RETAIN:	
1. In the past 6 months, have you ever been in a relationship where a sexual partner threatened you with violence? 2. In the past 6 months, have you ever been in a relationship where a sexual partner threw, broke, or punched things? 3. In the past 6 months, have you ever been in a relationship where you felt controlled by a sexual partner?	
Response options: Yes, No	

### Safety protocols

All participants were informed that responses to study measures are kept confidential within their respective sites and that data used for research purposes is de-identified. In CNICS, patients are informed, prior to answering questions, that their providers will see their responses. Patients in all studies were given the option of not answering any questions. PLWH in CNICS indicating any type of violence in the IPV-4 prompt a pager alert for an on-site social worker to check-in with the patient, same-day, on-site during their clinic visit, at which point they are evaluated and provided with resources if needed. For the PACTO and RETAIN studies, indication of IPV prompted an assessment in real time with the patient by a licensed psychologist who was part of the study team.

### Outcomes

We examined the association between psy-IPV and the following risk behaviors and symptoms: depressive symptoms, ART adherence, current use of methamphetamine/crystal, cocaine/crack, illicit opioids, marijuana, and alcohol, as well as current binge alcohol use. Depressive symptoms was defined as a score of ≥10 on the PHQ-9 [42, 43] in CNICS, and a score of ≥16 on the Center for Epidemiological Studies-Depression (CES-D-20) [44] measure in PACTO and RETAIN. Adherence to ART was measured as a percentage of HIV medication taken over the past 30 days [45]. Current alcohol use was defined as a score of ≥1 as measured by the AUDIT-C [46] and current binge drinking was defined as having had 5 or more drinks on one occasion using the AUDIT-C. Drug use was identified using the ASSIST [47] which includes methamphetamines, illicit opioids, cocaine/crack, sedatives, stimulants, hallucinogens, inhalants, and marijuana. We examined associations between psy-IPV and clinical outcomes including HIV care cascade steps, specifically ART use, detectable viral load, and CD4 count, as well as self-reported ART adherence.

### Statistical analysis

We performed statistical analysis using STATA version 14.2. We used logistic regression for analyses with binary variables and linear regression for analyses with continuous variables, to compare those who did and did not indicate psy-IPV. Observations were weighted by inverse probability of treatment weights (IPTW) to reduce the ratio of adjustment factors to outcomes in models; IPTW using propensity scores is a good alternative to control for confounding when there are seven or fewer events per confounder [48]. We generated propensity scores using logistic regression based on the model by Hernan et al. (2000) [49]. IPTWs were calculated using two different propensity score models to estimate the propensity of indicating psy-IPV: (1) Propensity of indicating psy-IPV was estimated adjusting for a limited set of covariates including age, site, and race/ethnicity; (2) Propensity of indicating psy-IPV was estimated by adjusting for a larger set of covariates including age, site, race/ethnicity, current substance use (methamphetamine, cocaine/crack, illicit opioids, marijuana, alcohol and binge alcohol use) and depressive symptoms. Age was modeled linearly. Propensity score density plots were created for both scores to assess adequacy of overlap between participants that reported psy-IPV and those that did not report psy-IPV. We performed sensitivity analyses in CNICS with propensity scores that also adjusted for physical IPV. We examined demographic characteristics among those with and without psy-IPV including age, sex, race/ethnicity, and sexual orientation. We examined psy-IPV and risk behaviors and symptoms as well as clinical characteristics including HIV care cascade steps. These associations were assessed using the limited IPTW, and associations except depressive symptoms and substance use were also assessed using the more complex IPTW. When age was compared between those with and without psy-IPV, the model was not weighted and simply adjusted for study and race since age was in both IPTWs. We examined the association of psy-IPV and HIV care cascade variables exclusively in the CNICS cohort, due to smaller sizes of the other studies. We note that due to discordant recall periods between the IPV measures administered by study sites, we did not perform formal mediation analyses.

A list of all statistical models that were run can be found in Supplemental Table 1.

## Results

### Study population

A total of 5950 PLWH were included in this study, with 564 (10%) indicating recent psy- IPV; CNICS contributed the largest number of participants (5195), followed by PACTO (408), and RETAIN (347) (Table 3). Of the 5950 participants, 18% were female sex at birth, and the mean age was 47 years. One percent of participants were transgender. Race and ethnicity varied across studies, with CNICS having 45% white, PACTO having 99% Hispanic, and RETAIN having 82% black participants. Depressive symptoms (25%) and substance use (up to 62%) were commonly reported. In CNICS, which assessed physical/sexual and psy-IPV, 50% of those indicating psy-IPV did not report physical/sexual IPV. PACTO/RETAIN did not have data on physical or sexual IPV within a harmonizable time window (asked lifetime only), hence, psy-IPV independent of physical/sexual IPV could not be assessed for those sites.

**Table 3:** Demographic and clinical characteristics of PLWH by study

Study	CNICS	PACTO^**a**^	RETAIN^**a**^	Total
**N**	5195	408	347	5950
**Any psy-IPV**	457 (9%)	71 (17%)	36 (10%)	564 (9%)
Felt controlled, but not threatened	194 (4%)	13 (3%)	2 (0.6%)	209 (4%)
Felt threatened, but not controlled	38 (0.7%)	18 (4%)	7 (2%)	63 (1%)
Felt both controlled and threatened	225 (4%)	40 (10%)	27 (8%)	292 (5%)
**Female**^**b**^	855 (16%)	94 (23%)	124 (36%)	1073 (18%)
**Age** (mean, SD)	47 (12)	46 (9)	46 (9)	47 (11)
**Race/ethnicity**				
White	2320 (45%)	1 (0%)	15 (4%)	2336 (39%)
Black	1811 (35%)	0 (0%)	286 (82%)	2097 (35%)
Hispanic	779 (15%)	405 (99%)	32 (9%)	1216 (20%)
Other/Unknown	285 (5%)	2 (0%)	14 (4%)	301 (5%)
**Heterosexual**	1434 (28%)	348 (85%)	173 (50%)	1955 (33%)
**Men who have sex with men**	3401 (66%)	41 (10%)	45 (19%)	3487 (60%)
**VL** **>** **400**	466 (9%)	192 (47%)	326 (94%)	984 (17%)
**CD4** (mean, SD)	668 (331)	540 (346)	181 (150)	631 (344)
**Depression**	926 (18%)	325 (80%)	264 (76%)	1515 (25%)
**ART use**	4827 (93%)	297 (73%)	191 (55%)	5315 (89%)
**ART adherence**^c^ (mean %, SD)	94 (14)	95 (16)	90 (21)	94 (14)
**Substance Use**				
Methamphetamine/crystal	446 (9%)	6 (1%)	3 (1%)	455 (8%)
Cocaine/crack	295 (6%)	230 (56%)	244 (70%)	769 (13%)
Illicit opioid	107 (2%)	186 (46%)	29 (8%)	322 (5%)
Marijuana	1514 (29%)	106 (26%)	118 (34%)	1738 (29%)
Alcohol	3319 (64%)	162 (40%)	224 (65%)	3705 (62%)
Binge alcohol	1677 (32%)	109 (27%)	158 (46%)	1944 (33%)

^a^PACTO and RETAIN were both STTR studies

^b^Birth sex

^c^Adherence defined as % of medication taken in past 30 days

Abbreviations: *ART*antiretroviral therapy; *IPV* intimate partner violence; *SD* standard deviation; *VL* viral load

### Associations with psychological IPV

Propensity score density plots (Supplemental Figs. 1 and 2) showed good overlap between participants reporting psy-IPV and those not reporting psy-IPV, confirming that IPTW is an appropriate method to control for confounding (Table 4; see Supplemental Tables 2, 3, and 4 for breakdowns by study site). Those reporting psy-IPV were on average 3 years younger (95% CI [2, 4], *p*-value < 0.001). In models using the limited IPTW, psy-IPV was significantly associated with HIV outcomes including lower odds of ART use (0.73 [0.55,0.97], *p* = 0.03), lower ART adherence (− 4.2 [− 5.9,-2.4], *p* < 0.001), and higher odds of having a detectable VL (1.43 [1.15,1.78], *p* = 0.001). Psy-IPV was also associated with higher odds of depressive symptoms (2.63 [2.18,3.18], *p* < 0.001) and greater odds of methamphetamine, cocaine/crack, illicit opioid, and marijuana use, with methamphetamine use having the largest odds ratio (2.98 [2.30,3.87], *p* < 0.001). Female sex at birth, alcohol use, binge alcohol use, sexual orientation, and CD4 count were not significantly associated with psy-IPV.

**Table 4:** Factors associated with psy-IPV. Inverse probability of treatment weights (IPTW) used based on propensity scores

	IPTW with propensity score including age, site, race/ethnicity only	IPTW with propensity score including age, site, race/ethnicity, substance use, depression
Characteristic	OR for IPV (95% CI),*p*-value	OR for IPV (95% CI),*p*-value
Female	1.20 (0.95,1.51),0.1	1.24 (0.96,1.60),0.1
VL > 400	1.43 (1.15,1.78),0.001	1.21 (0.94,1.57),0.1
Depression	2.63 (2.18,3.18),< 0.001	NA
ART use	0.73 (0.55,0.97),0.03	0.86 (0.62,1.18),0.3
Methamphetamine/crystal use	2.98 (2.30,3.87),< 0.001	NA
Cocaine/crack use	1.57 (1.24,1.99),< 0.001	NA
Illicit opioid use	1.56 (1.13,2.16),0.007	NA
Marijuana use	1.40 (1.15,1.70),0.001	NA
Alcohol use	1.04 (0.86,1.27),0.7	NA
Binge alcohol use	1.18 (0.98,1.43),0.09	NA
Heterosexual^a^	0.87 (0.71,1.06),0.2	0.87 (0.69,1.09),0.2
Outcome	Coeff for IPV (95%CI),p-value	Coeff for IPV (95%CI),p-value
CD4	13.1 (−20.0,46.3),0.4	−3.6 (−40.5,33.3),0.8
ART adherence (VAS)	−4.2 (−5.9,-2.4),< 0.001	−1.5 (− 3.1,-0.2),0.047
Age^b^	− 3.2 (− 4.1,-2.3),< 0.001	NA

Abbreviations: *ART* antiretroviral therapy; *IPV* intimate partner violence; *VAS* visual analog scale; *VL* viral load

Note: due to missing data, N for larger propensity score was 5422 with 490 IPV

^a^Homosexual or bisexual reference

^b^Not weighted, adjusted for age and race/ethnicity

Using the full IPTW, which additionally adjusted for depressive symptoms and substance use, effect sizes for psy-IPV were uniformly smaller and psy-IPV was no longer associated with most outcomes (e.g. ART use, CD4 count) except for ART adherence (− 1.5 [− 3.1,-0.2], *p* = 0.047). After including depressive symptoms and substance abuse in the propensity score, female sex was significant for psy-IPV in PACTO and RETAIN (Table 4, Supplement Tables 2, 3 and 4).

In CNICS, 50% of patients who indicated psy-IPV did not also indicate physical or sexual IPV. Our sensitivity analysis on the effects of psy-IPV adjusting for physical and sexual IPV in CNICS (Table 5) showed that when using a propensity score that includes age, site, race/ethnicity, and physical/sexual IPV, that psy-IPV was associated with having a viral load > 400 [1.82 (1.20,2.76), *p* = 0.005], depressive symptoms [3.21 (2.42,4.27), *p* < 0.001], lower odds of using ART [0.52 (0.32,0.87),0.01], and higher odds of methamphetamine/crystal, cocaine/crack, and illicit opioid use [2.25 (1.57,3.24), *p* < 0.001; 2.01 (1.25,3.22),*p* = 0.004; 2.06 (1.08,3.95),*p* = 0.03, respectively]. Additionally, after adjusting for site, race/ethnicity, and physical/sexual IPV, those who indicated psy-IPV were on average 1.8 years younger (− 3.1,-0.6), *p* = 0.005 than those who did not. When adding substance use and depressive symptoms to the propensity score analysis, these associations were no longer present.

**Table 5:** Association of any IPV (psychological) with demographic and clinical characteristics in CNICS. Inverse probability of treatment weights (IPW) used based on propensity scores including physical IPV. Note: due to missing data, N for larger propensity score was 4646 with 379 IPV

	IPW with propensity score including age, site, race/ethnicity, physical/sexual IPV only	IPW with propensity score including age, site, race/ethnicity, substance use, depression, physical/sexual IPV
Characteristic	OR for IPV (95%CI),*p*-value	OR for IPV (95%CI),p-value
Female	0.80 (0.54,1.20),0.3	0.67 (0.43,1.05),0.08
VL > =400	1.82 (1.20,2.76),0.005	1.45 (0.88,2.40),0.1
Depression	3.21 (2.42,4.27),< 0.001	NA
ART use	0.52 (0.32,0.87),0.01	0.87 (0.48,1.56),0.6
Methamphetamine/crystal use	2.25 (1.57,3.24),< 0.001	NA
Cocaine/crack use	2.01 (1.25,3.22),0.004	NA
Opioid use	2.06 (1.08,3.95),0.03	NA
Marijuana use	1.20 (0.90,1.60),0.2	NA
Alcohol use	1.29 (0.96,1.75),0.09	NA
Binge alcohol use	1.24 (0.94,1.64),0.1	NA
Heterosexual^a^	0.78 (0.56,1.09),0.1	0.67 (0.46,0.98),0.04
Outcome	Coeff for IPV (95%CI),p-value	Coeff for IPV (95%CI),p-value
CD4	14.6 (−29.4,58.6),0.5	8.5 (−40.5,57.5),0.7
ART adherence (VAS)	−2.6 (− 4.6,-0.7),0.007	−0.5 (− 2.0,1.0),0.5
Age^b^	−1.8 (−3.1,-0.6),0.005	NA

Abbreviations: ART-antiretroviral therapy; IPV-intimate partner violence; VAS-visual analog scale; VL-viral load

^a^Homosexual or bisexual reference

^b^ Not weighted, adjusted for age and race/ethnicity

In analyses that stratified by viral load, psy-IPV was significantly associated with depressive symptoms and methamphetamine/crystal use regardless of viral load [depressive symptoms 2.96 (2.37, 3.71), *p* < 0.001 undetectable, 1.81 (1.19,2.74),*p* = 0.005 detectable; methamphetamine/crystal use [2.96 (2.20,4.00),*p* < 0.001 undetectable, 3.00(1.75,5.14),*p* < 0.001 detectable], whereas psy-IPV was significantly associated with cocaine/crack [2.14(1.57, 2.93),*p* < 0.001], illicit opioid [2.19(1.42,3.35),*p* < 0.001], and marijuana use [1.55(1.24,1.94),*p* < 0.001] only in those with undetectable viral loads (Table 6).

**Table 6:** Associations between psy-IPV and outcomes, stratified by detectable VL. Inverse probability of treatment weights (IPTW) used based on propensity scores

	IPW with propensity score including age, site, race/ethnicity only – Undetectable VL	IPW with propensity score including age, site, race/ethnicity only – Detectable VL
Characteristic	OR for IPV (95%CI),p-value	OR for IPV (95%CI),p-value
Depression	2.96 (2.37,3.71),< 0.001	1.81 (1.19,2.74),0.005
Methamphetamine/crystal use	2.96 (2.20,4.00),< 0.001	3.00 (1.75,5.14),< 0.001
Cocaine/crack use	2.14 (1.57,2.93),< 0.001	0.82 (0.55,1.21),0.3
Opioid use	2.19 (1.42,3.35),< 0.001	0.83 (0.50,1.37),0.5
Marijuana use	1.55 (1.24,1.94),< 0.001	0.95 (0.62,1.44),0.8
Alcohol use	0.99 (0.78,1.24),0.9	1.43 (0.95,2.16),0.08
Binge alcohol use	1.15 (0.92,1.43),0.2	1.33 (0.90,1.96),0.2

Abbreviations: *IPV* intimate partner violence, *VL* viral load

## Discussion

We found that psy-IPV was common among PLWH (ranging from 9 to 17% across studies) and associated with having a detectable viral load (VL > 400) among PLWH. We also found that psy-IPV was associated with depressive symptoms and most types of substance use, with the exception of non-binge alcohol use. In a sensitivity analysis, even after adjusting for physical and sexual IPV, we found psy-IPV was associated with lower odds of ART use and poorer adherence. These associations were not present in analyses that also adjusted for substance use and depressive symptoms, suggesting that substance use and depressive symptoms may be on the causal pathway between psy-IPV and HIV outcomes and act as mediators. This underscores the need to address these areas simultaneously in clinical settings.

Psy-IPV did not vary across most demographic groups, with the exception that younger PLWH were more likely to indicate it than their older counterparts. Notably, psy-IPV was just as likely to be present in groups that are traditionally less-likely to be screened for IPV, such as men. This relative lack of demographic variance in reporting psy-IPV highlights the importance of screening all patients in HIV care for IPV, regardless of gender, race, or sexual orientation.

A major limitation of the majority of prior work is the lack of controlling for physical and sexual IPV in order to determine if psy-IPV has an independent effect on health outcomes in the absence of physical and/or sexual IPV. Prior work in the general population attempting to parse out the effects of psy-IPV from physical or sexual IPV has shown that, in a large sample of women, compared to those that had not experienced IPV of any kind, women indicating psy-IPV in the absence of physical or sexual IPV reported poorer physical and mental health as well as higher likelihood of having had a sexually transmitted infection, and higher likelihood of reporting physical symptoms [30].

Our findings also echo those of Jewkes et al. [25] which found emotional abuse to be associated with decline in cellular immunity, and Shafer et al. [24] which found psy-IPV to be associated with CD4 < 200 and detectable viral load, and ‘being threatened by a partner’ to be associated with these factors as well as a high no-show rate in HIV care. Our study builds on these using a larger multi-site sample, measures that were validated across sub-populations of PLWH, and the use of patient self-report to elicit data rather than the use of structured interviews, the former of which is known to reduce response bias in IPV reporting [50].

Given a) the suspected impact of psy-IPV on health outcomes even in the absence of physical or sexual IPV, b) the high prevalence of psy-IPV among PLWH compared to other types of IPV, and c) that those reporting psy-IPV in the absence of physical or sexual IPV have shown to be more at risk for continued exposure to psy-IPV [29], we stress the importance of IPV screening that includes a psychological dimension. Psy-IPV remained associated with ART adherence after controlling for depressive symptoms and substance use, adding another important dimension for understanding and addressing factors contributing to suboptimal adherence. Screening for IPV has improved provider documentation of identification of IPV and referrals [51]. In one large study, computer-based screening for IPV increased rates of IPV discussion, disclosure, and services provided in emergency clinic settings [52]; in another study, it was far more effective than usual care in identifying IPV: 19% of women who were administered the screening indicated IPV vs. 1% among the controls (*n* = 1005) [53]. Relative to face-to-face screening, computer-based screening has been shown to be more effective in identifying IPV and to be as effective as clinician interview in terms of disclosure, patient comfort, and time spent screening [50], in addition, it has shown to be well tolerated by patients and clinicians [50], even preferred to in-person questioning [54]. Importantly, computer-based screening has not been shown to increase prevalence of IPV [54].

For these reasons, we recommend same-day, pre-visit, computer-based, patient-administered PRO screening for IPV including both physical/sexual and psy-IPV in HIV care for patients of all genders, accompanied by measures of substance use, depressive symptoms, and ART adherence, as a standard of care in HIV care settings. Further, we recommend additional research into other factors that affect PLWH that may influence susceptibility to psy-IPV. These include environmental factors, such as housing status, financial need, experience of HIV-related stigma, and social support, as well as person-level factors such as changes to cognitive function or depression. Finally, we recommend further investigation into possible mediating factors that may affect the relationship between psy-IPV and HIV-related outcomes in order to inform future interventions as well as additional research to better understand the mechanisms by which psy-IPV impacts these outcomes.

### Strengths

The multi-site nature of this study yielded a demographically and geographically diverse sample of study participants.

### Limitations

Study data was heavily weighted towards CNICS (87%). Also, since data for physical and sexual IPV was not harmonizable across both consortia, we were only able to examine outcomes of psy-IPV in the context of known presence or absence of physical and sexual violence for PLWH in CNICS. The fact that participating PLWH in the study were at least somewhat engaged in care may have introduced selection bias, as those out of care may experience greater psychological abuse and more adverse health outcomes. We note that while all three study populations are care-based groups, study criteria varied, as did the IPV assessment including the platform and setting, which, while increasing generalizability, may influence interpretability of the results. Finally, due to the cross-sectional nature of the study, we were not able to determine the directionality of associations such as between substance use and psy-IPV.

## Conclusion

Psychological IPV, even in the absence of physical or sexual IPV, is associated with poorer health behaviors, including higher rates of substance use, lower ART adherence, and worse virologic outcomes. Patient-reported, self-administered measures of IPV show promise in helping providers identify IPV. In outpatient HIV care, such measures should include items querying psychological violence, and be accompanied by measures of depressive symptoms, adherence, and substance use.

## Supplementary Information

**Additional file 1.**

**Additional file 2.**

## Data Availability

Data and materials are archived by both the University of Washington Data Coordinating Center and NIDA. They can be made available upon reasonable request, with a concept proposal and fully executed data use agreement (due to the sensitivity of the data). Interested investigators can email jacd@uw.edu for more details.

## Abbreviations

ART: Antiretroviral therapy
ASSIST: Alcohol, Smoking and Substance Involvement Screening Test
AUDIT-C: Alcohol Use Disorders Identification Test
CNICS: Centers for AIDS Research Network of Integrated Clinical Systems
IPTW: Inverse probability treatment weight
IPV: Intimate partner violence
MSM: Men who have sex with men
PACTO: Proveyendo Acceso a Cuidado y Tratamiento
PLWH: Patients living with HIV
Psy-IPV: Psychological intimate partner violence
STTR: Seek, Test, Treat, Retain initiative
