# Supplementary material for: Correlates of psychological intimate partner violence with HIV care outcomes on patients in HIV care
Source: BMC Public Health. 2021 Oct 9;21:1824. doi: 10.1186/s12889-021-11854-x (PMC8502266; doi:10.1186/s12889-021-11854-x)

Supplemental Figure 1. Propensity score plot with limited covariates.


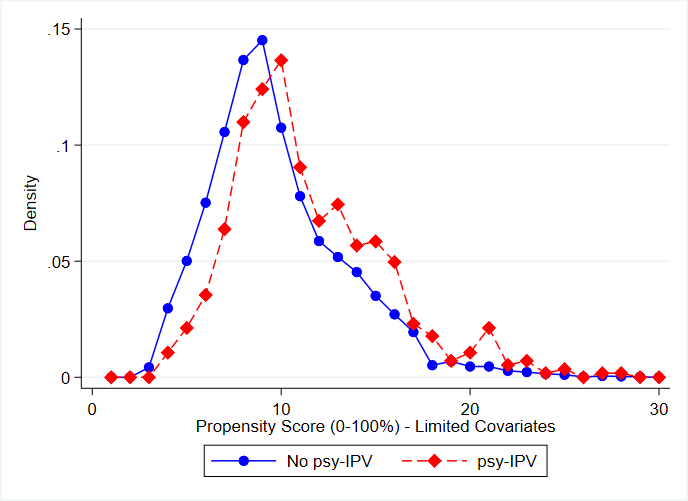


Supplemental Figure 2. Propensity score plot with full covariates.
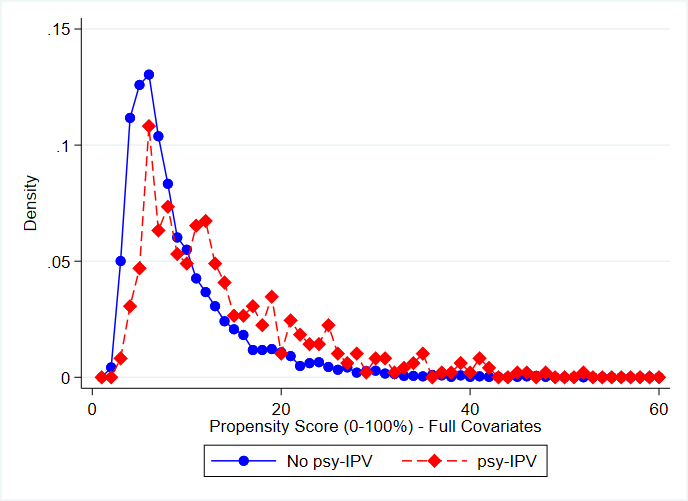

Supplement: Supplementary file 1 — Additional file 1. [file 12889_2021_11854_MOESM1_ESM.docx]
