# Supplementary material for: Correlates of psychological intimate partner violence with HIV care outcomes on patients in HIV care
Source: BMC Public Health. 2021 Oct 9;21:1824. doi: 10.1186/s12889-021-11854-x (PMC8502266; doi:10.1186/s12889-021-11854-x)
Supplement: Supplementary file 2 — Additional file 2. [file 12889_2021_11854_MOESM2_ESM.docx]

Supplemental Table 1. Description of included data and adjustment factors in tables with models.

| Table | Included data | IPTW variables |
| --- | --- | --- |
| 2, column 2 | all | basic |
| 2, column 3 | all | basic+substances+depression |
|  |  |  |
| 3, column 2 | CNICS | basic+physical IPV |
| 3, column 3 | CNICS | basic+substances+depression+physical IPV |
|  |  |  |
| 4, column 2 | Undetectable VL | basic |
| 4, column 3 | Detectable VL | basic |
|  |  |  |
| Supplemental 1, column 2 | CNICS | basic |
| Supplemental 1, column 3 | CNICS | basic+substances+depression |
|  |  |  |
| Supplemental 2, column 2 | PACTO | basic |
| Supplemental 2, column 3 | PACTO | basic+substances+depression |
|  |  |  |
| Supplemental 3, column 2 | RETAIN | basic |
| Supplemental 3, column 3 | RETAIN | basic+substances+depression |

Supplement Table 2. Associations between of any psy-IPV with demographic and clinical characteristics in CNICS. Inverse probability of treatment weights (IPTW) used based on propensity scores

|  | IPTW with propensity score including age, site, race/ethnicity only | IPTW with propensity score including age, site, race/ethnicity, substance use, depression |
| --- | --- | --- |
| Characteristic | OR for IPV (95%CI),p-value | OR for IPV (95%CI),p-value |
| Female | 0.99 (0.75,1.32),1.0 | 0.98 (0.71,1.36),0.9 |
| VL >400 | 1.89 (1.42,2.52),<0.001 | 1.29 (0.88,1.88),0.2 |
| Depression | 3.50 (2.82,4.33),<0.001 | NA |
| ART use | 0.58 (0.40,0.86),0.006 | 0.86 (0.54,1.39),0.5 |
| Methamphetamine/crystal use | 3.04 (2.32,3.98),<0.001 | NA |
| Cocaine/crack use | 2.42 (1.74,3.36),<0.001 | NA |
| Illicit opioid use | 2.84 (1.73,4.68),<0.001 | NA |
| Marijuana use | 1.50 (1.21,1.86),<0.001 | NA |
| Alcohol use | 1.04 (0.84,1.30),0.7 | NA |
| Binge alcohol use | 1.17 (0.95,1.44),0.1 | NA |
| Heterosexual* | 0.86 (0.68,1.10),0.2 | 0.80 (0.60,1.06),0.1 |
|  |  |  |
|  | Coeff for IPV (95%CI),p-value | Coeff for IPV (95%CI),p-value |
| CD4 | 6.5 (-28.6,41.6),0.7 | -6.8 (-46.7,33.2),0.7 |
| ART adherence (VAS) | -5.0 (-6.9,-3.1),<0.001 | -2.0 (-3.7,-0.4),0.016 |
| Age** | -3.4 (-4.4,-2.3),<0.001 | NA |

Abbreviations: ART-antiretroviral therapy; IPV-intimate partner violence; VAS-visual analog scale; VL-viral load.

Note: due to missing data, N for larger propensity score was 4688 with 385 IPV

*Homosexual or bisexual reference

** Not weighted, adjusted for age and race/ethnicity

Supplement Table 3. Associations of any psy-IPV with demographic and clinical characteristics in PACTO. Inverse probability of treatment weights (IPTW) used based on propensity scores.

|  | IPTW with propensity score including age, site, race/ethnicity only | IPTW with propensity score including age, site, race/ethnicity, substance use, depression |
| --- | --- | --- |
| Characteristic | OR for IPV (95%CI),p-value | OR for IPV (95%CI),p-value |
| Female | 2.01 (1.15,3.51),0.015 | 2.28 (1.29,4.04),0.005 |
| VL >400 | 0.99 (0.59,1.68),1.0 | 1.05 (0.61,1.79),0.9 |
| Depression | 0.91 (0.48,1.73),0.8 | NA |
| ART use | 1.46 (0.80,2.68),0.2 | 1.26 (0.68,2.36),0.5 |
| Methamphetamine/crystal use | 3.87 (0.75,19.9),0.1 | NA |
| Cocaine/crack use | 0.92 (0.54,1.56),0.8 | NA |
| Illicit opioid use | 0.92 (0.54,1.55),0.7 | NA |
| Marijuana use | 1.26 (0.72,2.23),0.4 | NA |
| Alcohol use | 1.23 (0.73,2.08),0.4 | NA |
| Binge alcohol use | 1.61 (0.92,2.81),0.09 | NA |
| Heterosexual* | 0.71 (0.36,1.41),0.3 | 0.68 (0.33,1.37),0.3 |
|  |  |  |
| Outcome | Coeff for IPV (95%CI),p-value | Coeff for IPV (95%CI),p-value |
| CD4 | 73.3 (-28.2,174.8),0.2 | 90.2 (-19.7,200.0),0.1 |
| ART adherence (VAS) | -0.8 (-5.5,3.9),0.7 | 0.1 (-4.2,4.3),1.0 |
| Age** | -2.2 (-4.4,0.1),0.06 | NA |

Abbreviations: ART-antiretroviral therapy; IPV-intimate partner violence; VAS-visual analog scale; VL-viral load.

Note: due to missing data, N for larger propensity score was 403 with 71 psy-IPV

*Homosexual or bisexual reference

** Not weighted, adjusted for age and race/ethnicity

Supplement Table 4. Association of psy-IPV with demographic and clinical characteristics in RETAIN. Inverse probability of treatment weights (IPTW) used based on propensity scores.

|  | IPTW with propensity score including age, site, race/ethnicity only | IPTW with propensity score including age, site, race/ethnicity, substance use, depression |
| --- | --- | --- |
| Characteristic | OR for IPV (95%CI),p-value | OR for IPV (95%CI),p-value |
| Female | 3.91 (1.88,8.14),<0.001 | 5.01 (2.20,11.42),<0.001 |
| VL >400 | 0.72 (0.19,2.76),0.6 | 1.03 (0.21,5.11),1.0 |
| Depression | 1.74 (0.64,4.74),0.3 | NA |
| ART use | 0.63 (0.30,1.31),0.2 | 0.60 (0.25,1.44),0.3 |
| Methamphetamine/crystal use | 3.16 (0.28,36.0),0.4 | NA |
| Cocaine/crack use | 1.11 (0.47,2.64),0.8 | NA |
| Illicit opioid use | 0.60 (0.17,2.12),0.4 | NA |
| Marijuana use | 0.50 (0.22,1.15),0.1 | NA |
| Alcohol use | 0.90 (0.43,1.89),0.8 | NA |
| Binge alcohol use | 0.99 (0.48,2.02),1.0 | NA |
| Heterosexual* | 0.53 (0.22,1.24),0.1 | 0.65 (0.23,1.84),0.4 |
|  |  |  |
| Outcome | Coeff for IPV (95%CI),p-value | Coeff for IPV (95%CI),p-value |
| CD4 | 18.1 (-30.6,66.9),0.5 | 13.5 (-33.2,60.3),0.6 |
| ART adherence (VAS) | 9.9 (6.1,13.7),<0.001 | 8.8 (4.1,13.6),<0.001 |
| Age** | -3.1 (-6.0,-0.2),0.04 | NA |

Abbreviations: ART-antiretroviral therapy; IPV-intimate partner violence; VAS-visual analog scale; VL-viral load.

Note: due to missing data, N for larger propensity score was 328 with 34 psy-IPV

*Homosexual or bisexual reference

** Not weighted, adjusted for age and race/ethnicity
